# Hard stop: reestablishing the significance of abstinence in the treatment of late stage ultra-processed food addiction

**DOI:** 10.3389/fpsyt.2024.1488769

**Published:** 2024-11-13

**Authors:** Vera I. Tarman

**Affiliations:** ^1^ Renascent Foundation, Toronto, ON, Canada; ^2^ University of Toronto, Toronto, ON, Canada

**Keywords:** ultra-processed food addiction, ultra-processed food addiction treatment, abstinence, harm reduction, Koob’s model of addiction

## Abstract

Addiction is a complex neurobiological disorder characterized by compulsive drug-seeking and use despite harmful consequences. While abstinence-based approaches have long been the cornerstone of addiction treatment, recent years have seen legitimate challenges from harm reduction clinicians, and within the food addiction realm, the eating disorder treatment practitioners. This perspective emphasizes the role of abstinence in food addiction recovery using the Koob model and its concept of hyperkatifeia despite these reservations. However, further research is essential before abstinence can be recommended. We need to 1) identify what qualifies as abstinence in relation to ultra-processed food, 2) clarify suitable situations and disease progression for optimal implementation of this approach, 3) provide clear guidelines when it is harmful, and 4) conduct clinical studies to confirm the effectiveness of this strategy for long-term recovery from late-stage food addiction.

## 1 Introduction

Addiction is a complex neurological condition defined by compulsive engagement in substance use or behaviours, regardless of negative outcomes. It affects millions worldwide, including those struggling with food addiction, where it has been estimated to be at least 14% of the adult population (1). While abstinence has long been a cornerstone of addiction treatment, its mechanisms and importance are not always understood, especially in ultra-processed food addiction, where the most effective and “best treatment” practices are still being established. Indeed, the various challenges involved in conceptualizing abstinence can make it appear to be an unachievable goal. As a result, it may seem expedient to explore more nuanced alternatives (2).

This perspective aims to reestablish the significance of abstinence as a key intervention in addiction recovery, specifically for food addiction. I will use the Koob model of addiction, which portrays addiction as a progressive condition with identifiable stages. It will focus on the “hyperkatifeic moment,” a critical point where the individual engages in the addictive behavior to avoid negative emotions rather than to seek pleasure (3). This moment marks a pivotal juncture where the addictive substance or behaviour has gained an overwhelming influence over the brain, and any moderate use within this highly sensitized system will most likely lead to a continued progression of the disease. There is no turning back to a pre-disease state, where moderate use is possible. Only abstinence can halt this progression.

By applying this conceptual framework, this article takes the position that abstinence is essential to treating individuals in the advanced stages of food addiction. Research substantiates the validity of this position. There are now a growing number of clinical studies that show that a low-carb keto diet, which includes the cessation of ultra-processed foods, are beneficial (4–7).

Even though it may be challenging, it is worthwhile to persist in establishing abstinence as a valid treatment approach. While emphasizing the importance of abstinence, this discussion will also address alternative perspectives, particularly harm reduction strategies, which may be more appropriate in certain scenarios (8). I will explore the critical distinction between food addiction and eating disorders, highlighting the potential dangers of applying abstinence-based approaches to individuals with eating disorders. I will also examine the practical considerations and challenges involved in applying an abstinence-based approach to food addiction treatment. This will include defining the parameters of abstinence and determining the optimal timing for implementing an abstinence-focused intervention, considering the different stages of the food addiction progression (9).

Ultimately, this work aims to emphasize the pivotal role of abstinence in sustaining long-term recovery for some who suffer from food addiction while acknowledging the need for harm reduction treatment strategies. By providing a comprehensive examination of the mechanisms and importance of abstinence, this perspective seeks to contribute to the ongoing discussion about effective treatment strategies for food addiction and highlight areas for future research.

## 2 Harm reduction: a paradigm shift

Past treatment paradigms targeting addiction have favoured abstinence over more moderate approaches, driven by the belief that complete avoidance of the addictive substance or behavior is the only path to true recovery. Indeed, the 12-step paradigm and well-known residential treatment programs such as Hazelden and Betty Ford have historically made abstinence the mainstay of their recovery approach (10–13). Pioneer clinicians in food addiction, such as Phil Werdell, have followed this tradition of promoting abstinence as the best treatment choice, despite the lack of sufficiently available scientific (14). However, in recent decades, a competing philosophy known as “harm reduction” has gained traction, particularly in substance abuse disorders like opioid addiction. Harm reduction seeks to minimize the negative consequences of addictive behaviours rather than demand total abstinence. There is abundant research that proves that this is the preferred and more successful model for addiction treatment (15, 16) as well as in food addiction specifically (4–7).

Food addiction is an emerging area of scientific inquiry. The literature suggests that the addictive nature of certain foods and the trajectory of food addiction mirror the patterns observed in drug and alcohol addiction (17). While research now suggests ultra-processed foods can be addictive, there is still no consensus on how this addiction manifests as a recognized clinical syndrome. This is a burgeoning area of research, and the understanding of how to conceptualize and define this condition properly is still in the early phases of development (13). Developing reliable methods for diagnosing and treating food addiction is still a work in progress (6, 18, 19).

Given this uncertainty surrounding the clinical syndrome of food addiction, the role of abstinence in its treatment is especially unclear. This lack of consensus has been evident for years among individuals attempting to find help for their problematic overeating. Diverse approaches adopted by the multiple 12-step programs, such as Overeaters Anonymous, Food Addicts in Recovery Anonymous, or Grey Sheeters Anonymous, all addressing food addiction, for example, have led to varying definitions of abstinence and different self-help practices. For some, it has meant restricting food groups, such as sugar and flour. For others, it has included portion control and defining specific mealtimes to avoid compulsive eating behaviors. In many peer-based groups, sobriety is determined “between you and your sponsor”. Sobriety is important, but to an outside observer, various displays of abstinence could be interpreted as a blending of moderation and abstinence (as in Overeater’s Anonymous ‘dignity of choice’ directive).

On the clinical front, the definition of abstinence in ultra-processed food addiction remains equally ambiguous. Should the therapist only recommend the cessation of refined carbohydrates or of all ultra-processed foods? Is addressing psychological and social cues a necessary part of the therapeutic mandate? Given the uncertainty surrounding the specific components that make food addictive, determining which foods or behaviours must be avoided has been challenging (20).

Abstaining from sugar or ultra-processed foods (UPFs) presents significant challenges in modern food environments. The ubiquity and availability of UPFs pose a major obstacle, as they make up a substantial portion of the diet in many developed countries. Cost and convenience play crucial roles in the difficulty of abstaining from UPFs. These products are often less expensive and more readily available than fresh, unprocessed alternatives, particularly in underserved communities (21). Social norms and cultural factors further complicate efforts to abstain from ultra-processed foods. As these foods have become deeply ingrained in many societies, avoiding them can lead to social isolation or difficulties in participating in communal eating experiences. There is a concern about the potential for developing restrictive eating habits when trying to eliminate entire food groups like fruit or dairy. There is apprehension that this approach could foster an unhealthy relationship with food or even cause nutritional imbalances if not carefully managed (22). Not surprisingly, abstinence as a treatment for food addiction is a frequently debated topic among clinicians. Many argue that a personalized, nuanced strategy that acknowledges the difficulty of total avoidance may be necessary to help individuals manage their food addiction sustainably (2, 23).

Harm reduction strategies, like those employed in substance addiction treatment, are recommended instead. Gradually reducing or substituting sugar consumption is suggested initially, as it can help mitigate the negative effects of halting. For instance, individuals can start by gradually reducing the amount of sugar in their coffee over time, allowing their taste preferences to adjust (24). Another common strategy involves substituting high-sugar foods with lower-sugar alternatives, like fruit, or natural sweeteners. Using low and no-calorie sweeteners can also be part of a harm reduction approach, as they can help reduce overall calorie and carbohydrate intake while still satisfying sweet cravings (25). Mindful eating practices is another strategy which involves paying close attention to hunger cues, eating slowly to savor foods. This can lead to greater satisfaction with smaller portions of sweet “treats”. Finally, ensuring adequate sleep and managing stress through relaxation techniques or exercise can help reduce sugar cravings and, thus, emotional eating (25). A harm reduction approach recognizes the inherent challenges of complete abstinence in the modern food environment and seeks to empower individuals by making gradual, sustainable changes that can minimize the negative consequences of their addictive behaviors. As we will explore further in subsequent sections, these approaches may be highly effective in addressing the initial phases of food addiction, thus clearly have a place in the treatment of food addiction approaches.

## 3 Eating disorders and food addiction

It’s crucial to differentiate between food addiction and eating disorders, particularly binge eating and bulimia, as the early signs of each condition can mimic each other (26). Food addiction is characterized by loss of control of overeating, continued use despite negative consequences, and intense cravings, attributes that resemble substance use disorders. Its presentation typically involves compulsively overeating large amounts of food. In contrast, eating disorders such as bulimia nervosa or binge eating disorder involve complex psychological factors that go beyond the addictive eating behaviors observed in food addiction. Despite the distinct underlying causes, food addiction and eating disorders exhibit considerable overlap in their outward manifestations, making it challenging to determine which condition is the primary diver of the problematic eating behaviors (27).

Applying food addiction treatment models to eating disorders carries significant risks. Eating disorders often require a more nuanced approach to food intake, including the potential inclusion of ultra-processed foods. The decision of what to eat depends on the nutritional value, rather than the addictive or triggering nature of the food. The emphasis in treatment should be on addressing the underlying psychological factors, rather than solely focusing on the food itself, which is merely a symptom of the deeper distress. Enforcing strict abstinence, as done in addiction treatment, might trigger or exacerbate eating disorder symptoms (28). Individuals with eating disorders are already prone to food restriction, which can further intensify their unhealthy preoccupation with food and body image.

Despite these challenges that suggest a harm reduction-oriented approach may be judicious, I will show, through the lens of the Koob addiction model and the concept of hyperkatifeia, why abstinence is critical for long-term recovery, particularly for those in the advanced stages of food addiction.

## 4 The Koob model of addiction: a discussion

Koob’s model of addiction describes addiction as a progressive disease with stages ranging from experimentation to full-blown addiction (3). As the brain becomes desensitized to dopamine, repeated exposure to addictive substances causes changes in the brain that drive this progression toward tolerance and dependence (29). A critical point in this process, called “hyperkatifeia,” marks the shift from using substances for pleasure to using them to avoid discomfort. At this stage, the brain’s reward system is dysregulated, essentially “hijacked” by the addictive substance, resulting in increased cravings and a loss of control over substance use, despite the negative consequences—the hallmarks of full-blown addiction (30).

Hyperkatifeia involves several key mechanisms:

### 4.1 Tolerance

With prolonged drug use, individuals may develop a tolerance for the rewarding effects of the addictive substance or behavior. They require larger amounts or more frequent engagement to achieve the desired effect. This occurs because of the downregulation of dopamine 2 receptors to stabilize the impact of the dopamine surplus when using addictive substances.

### 4.2 Sensitization

Repeated exposure to addictive substances or behaviors can paradoxically lead to an increased sensitivity to their rewarding effects. This occurs because the remaining dopamine receptors attempt to respond to the downregulation by becoming highly sensitized. Part of receptor sensitization is caused by the buildup of transcription factor delta Fos B, which makes cravings stronger and increases the desire to engage in addictive behavior. Delta Fos B-mediated sensitization can persist long after drug use has ceased, contributing to the chronic nature of addiction and vulnerability to relapse (31).

### 4.3 Withdrawal

When the addictive substance or behavior is removed, individuals may experience withdrawal symptoms, which can be experienced as profound discomfort on both physical and psychological levels. Tolerance has produced a “new normal” and when repeated use subsides, the brain responds to the deficit of dopamine with great distress, leading to increased motivation to keep using.

### 4.4 Conditioned “learned” behavior forms when the brain repeatedly links environmental cues with drug rewards

The repeated pairing of environmental cues with the rewards of the addictive substance or behavior generates triggers that evoke cravings and physiological reactions, even when the addictive substance is not present. This process strengthens with prolonged use, creating powerful associations that can lead an individual to relapse when encountering these triggers.

### 4.5 Frontal lobe dysregulation

In individuals with advanced addictions, the frontal lobe, which oversees critical executive functions such as impulse control, decision-making, and behavioral regulation, becomes dysregulated. The impaired ability to control impulses contributes to the persistent pursuit of addictive behavior, even in the face of negative consequences. Therefore, attempts at moderating the addictive substance are ineffective, as the frontal lobe’s capacity for self-regulation and impulse control is severely compromised (32).

These profound neurological changes define hyperkatifeia. They explain how the compulsive drive to use the addictive substance becomes too powerful, overwhelming the individual’s willpower and capacity for controlled, moderate consumption. They illustrate why abstinence is critical for individuals to achieve recovery.

First, abstinence interrupts the increasing progression of the addiction. It allows for the re-establishment of neural pathways and the restoration of appropriate dopamine receptor sensitivity, which is essential to recovering the brain’s reward system (33, 34) and reducing the intense cravings associated with addiction. This process of neural reset is crucial for maintaining long-term recovery. Prolonged abstinence is also linked to substantial enhancements in cognitive abilities, such as decision-making, impulse regulation, and executive functioning. Research has shown that abstinence can lead to increases in neuronal gray matter volume and improvements in neuronal white matter integrity, which results in better performance on tasks measuring attention, working memory, and cognitive flexibility (35). Abstinence is also more likely to lead to better emotional regulation, as individuals learn to cope with stress and negative emotions without relying on addictive substances or behaviors.

It is important to note that some neuroadaptations associated with addiction may persist even after abstinence, underscoring the chronic and progressive nature of addiction (36). For many individuals, the potential for relapse remains a persistent concern, requiring ongoing vigilance to maintain their recovery.

## 5 Abstinence in food addiction treatment

Given the dynamic nature of abstinence, a comprehensive definition of abstinence in ultra-processed food addiction is the personalized cessation of problematic eating behaviors and consumption of trigger foods, typically including ultra-processed items high in sugar, refined flour, or unhealthy fats. For many, it can include refraining from foods that are even minimally processed, if they are triggering to the individual, such as diary, grain, nuts. It exists on a spectrum from partial (harm reduction) to complete “hard stop” abstinence, tailored to an individual’s biological, psychological, and social factors. The definition of abstinence is dynamic, context-dependent, and aligned with the person’s recovery goals and stage of addiction.

Abstinence in food addiction is best perceived as a nuanced and flexible concept tailored to the individual’s stage of addiction. The progressive nature of food addiction requires a personalized approach to defining abstinence, which can vary significantly from person to person. Ultra-processed foods, known for their dopaminergic effects, can trigger addictive responses in some individuals, while others may tolerate limited consumption without issue. For individuals in the early stages of ultra-processed food addiction, abstinence may resemble a harm reduction strategy. This may include controlling the consumption of addictive foods, such as sugar and ultra-processed items, thus adopting a gradual abstinence of problematic food. Those who have not yet developed a significant tolerance might manage occasional indulgences in these foods.

As the addiction progresses, the definition of abstinence becomes more stringent. Substituting sugar with sweeteners and permitting select ultra-processed foods may be effective approaches. However, for those with advanced food addiction, complete abstinence from highly palatable and ultra-processed foods is often necessary (13, 37). Prolonged exposure to these foods can mimic addictive substances’ effects on the brain’s reward system, leading to tolerance, sensitization, and withdrawal symptoms (38). For individuals who have surpassed the hyperkatifeia stage, abstinence is best defined as the cessation of all foods that significantly activate the reward circuitry. Abstinence from ultra-processed foods alone may not suffice. Abstinence may involve consuming only unprocessed or minimally processed foods or foods “with one ingredient.” Sweeteners can be problematic as they mimic the sweet taste and can activate cravings. The range of “safe” foods typically narrows in this late stage as trigger foods, even healthy ones like nuts, cheese, and peanut butter, increase. Individuals at this stage often require a dietician to help them navigate a healthy and non-triggering food plan.

A “hard stop” abstinence approach acknowledges that for individuals with end-stage food addiction, moderation or harm reduction strategies may be ineffective or even detrimental. At this stage, individuals must maintain ongoing vigilance in avoiding ultra-processed foods, as even a small amount, months or years later, can trigger a relapse (13, 37). This situation is akin to an individual in long-term sobriety from alcohol who resumes drinking after the introduction of just “one or two” drinks. Once hyperkatifeia has occurred, a complete cure from addiction is unlikely, but ongoing management through sustained remission is possible.

## 6 Abstinent based approaches in ultra-processed food addiction: considerations and challenges

The Koob model and the concept of hyperkatifeia provide a theoretical foundation for including abstinence-oriented treatment approaches, especially for individuals with advanced food addiction. Abstinence-based treatment programs target strategies to help individuals identify and avoid personal trigger foods and problematic behaviors, such as restricting or overeating, while developing healthier eating habits tailored to their specific stage of food addiction.

The precise diagnosis of the food addiction stage is crucial in determining the suitable level of abstinence (9). Imposing strict abstinence on individuals with early-stage food addiction could be counterproductive, causing undue psychological and social distress, potentially leading them to discontinue treatment. In these cases, a harm reduction approach would be more appropriate. Conversely, being too lenient would not benefit those with advanced food addiction. A personalized approach ensures that individuals receive the most effective care tailored to their specific needs and addiction stage. It is also important to consider individuals with eating disorders, as they will probably not benefit from an abstinence-based approach.

An abstinence approach must go beyond defining and implementing an abstinence food plan. With no “one-size-fits-all” definition of abstinence, it becomes imperative to provide further support. This begins by educating individuals about what an abstinence-based diet might encompass for them personally. Complete abstinence of ultra-processed foods may seem especially daunting, presenting significant practical challenges. Many are also unaware of which ultra-processed foods trigger their addictive behaviors. Eliminating entire food categories to avoid trigger foods can lead to nutritional deficiencies if not managed properly (28). It’s essential that abstinence-based approaches in food addiction recovery are coupled with comprehensive nutritional guidance to ensure that all dietary needs are met through whole, unprocessed foods (14).

The psychological impact of strict abstinence can be also substantial. For some individuals, especially those with a history of disordered eating (very common amongst people suffering from food addiction), rigid food rules may exacerbate anxiety or trigger obsessive thoughts about food (28). The fear of breaking abstinence can lead to heightened stress levels, potentially undermining overall mental health and well-being. Additionally, the social isolation that may result from avoiding food-centric events can contribute to feelings of loneliness and depression (2, 22). Thus, professional resources are integral. Food coaches and addiction counselors, as well as community and peer support, such as the myriads of 12-step support groups, are required to help individuals maintain abstinence and manage cravings over an extended period.

Recognizing the persistent and challenging nature of addiction highlights the critical role of relapse prevention strategies. Individuals must navigate a world where trigger foods are ubiquitous, often integral to social gatherings and cultural traditions. The constant exposure to food advertisements and the prevalence of ultra-processed foods in most retail environments further complicate adherence to abstinence (39). Individuals recovering from food addiction must be provided with essential skills, support, and resources to manage cravings productively, overcome triggering situations, and sustain abstinence over the long-term recovery process.

Achieving sustainable abstinence from ultra-processed foods is challenging due to their ubiquity, affordability, convenience, and aggressive marketing. Resisting these products requires significant effort, and support for abstinence must extend beyond the individual and their immediate network. Government intervention is crucial, including stricter regulations on ultra-processed foods similar to those applied to tobacco and alcohol. Such measures could include advertising restrictions, mandating plain packaging, taxation and limiting availability. These actions would help reduce the environmental pressure on individuals trying to abstain from ultra-processed foods in a marketing-saturated landscape.

In addressing the challenges of abstinence in food addiction recovery, it is important to take a nuanced and individualized approach. This may entail accommodating the definition of abstinence based on the individual’s stage of addiction. Initially, a harm reduction strategy may be appropriate, but once the hyperkatifeia threshold has been crossed, the treatment must shift to a more rigid, structured platform that requires a corresponding increase in the level of support provided.

## 7 Conclusion

The Koob model of addiction and the concept of hyperkatifeia provide a compelling framework for understanding the mechanism and importance of abstinence in addiction recovery, particularly in late-stage food addiction. Complete abstinence is the only means to interrupt the cycle of addiction and allow the brain to repair and reset, leading to improvements in cognitive function, emotional regulation, and overall well-being (33–35). Healthcare providers should reconsider the significance of abstinence in maintaining long-term recovery for those with advanced food addiction and refine treatment strategies that prioritize abstinence-based approaches. These strategies should be tailored to address the unique challenges of late-stage food addiction, including the identification and elimination of trigger foods, the development of alternative healthier habits, and the institution of relapse prevention strategies to ensure sobriety in the long term.

The insights gained from the Koob model emphasize the need for further research to diagnose and validate the different stages of food addiction accurately and develop customized “best treatment” practices tailored to each stage. It will also be necessary to explore alternative treatment options for those individuals who cannot achieve complete abstinence, despite the recognized significance of this approach (2). Research that concentrates on creating and validating new assessment tools, like scales or biomarkers, to measure the “hyperkatifeic moment” - the stage when moderation is no longer a practical long-term solution - would be highly beneficial (3).

In conclusion, it is my perspective that while abstinence in ultra-processed food addiction treatment presents significant challenges, its potential benefits warrant continued exploration and refinement. By acknowledging the neurobiological basis of addiction and the limitations of moderation in advanced cases, we can work towards more effective, evidence-based treatments. This approach not only addresses the immediate needs of patients with severe food addiction, but also contributes to the broader understanding of addiction as a complex, multifaceted condition requiring equally sophisticated interventions.
